# Bacterial proteomic profile of early colonizers on the acquired pellicle of patients with head and neck cancer

**DOI:** 10.1590/1807-3107bor-2026.vol40.055

**Published:** 2026-08-07

**Authors:** Talita Mendes Oliveira VENTURA, Júlia Thomazini PEREIRA, Ramilli Maria de OLIVEIRA, Larissa Tercília Grizzo THOMASSIAN, Nathalia Regina RIBEIRO, Even Akemi TAIRA, Vinícius Taioqui PELÁ, Aline Dionizio VALLE, Cássia Maria Fisher RUBIRA, Paulo Sérgio da Silva SANTOS, Marília Afonso Rabelo BUZALAF

**Affiliations:** (a) Univeridade de São Paulo – USP, Bauru School of Dentistry, Department of Biological Sciences, Bauru, SP, Brazil.; (b) Univeridade de São Paulo – USP, Bauru School of Dentistry, Department of Surgery, Stomatology, Pathology and Radiology, Bauru, SP, Brazil.

**Keywords:** Head and Neck Neoplasms, Radiotherapy, Dental pellicle, Biofilms, Proteomics

## Abstract

Radiotherapy is the cornerstone of head and neck cancer (HNC) treatment, but it has been frequently associated with changes in the acquired enamel pellicle (AEP), potentially affecting the early stages of dental biofilm formation. Thus, this study aimed to characterize the bacterial proteomic profiles of early colonizers adhering to the AEP in patients with HNC undergoing radiotherapy, with emphasis on radiation-induced changes that may influence oral biofilm development and oral health outcomes. AEP samples were collected from nine patients with HNC before radiotherapy (BRT), during radiotherapy (DRT), and after radiotherapy (ART), as well as from nine orally healthy individuals. Pellicles were obtained 120 min after formation and analyzed by shotgun label-free proteomics using nanoLC-ESI-MS/MS. Protein expression levels were compared between groups using the t test (p < 0.05). AEP showed elevated levels of bacterial proteins predominantly attributed to *Actinomyces*, mainly associated with DNA binding, chromatin organization, and glucose metabolism. The bacterial proteomic profile shifted markedly DRT, with exclusive detection of proteins involved in carbohydrate transport and metabolism, oxidative stress response, and protein folding, including several ATP-binding cassette transporters and glycolytic enzymes. Proteins related to stress tolerance, transcriptional regulation, and secretion systems were detected ART, many of them associated with bacterial species commonly linked to periodontal diseases, such as *Tannerella* and *Oribacterium*. Notably, *Cysteine synthase* was identified exclusively BRT in all comparisons, suggesting its potential as a biomarker. These findings indicate that radiotherapy induces dynamic changes in the AEP bacterial proteome, potentially favoring dysbiotic biofilm development and increasing susceptibility to oral diseases in patients with HNC.

## Introduction

Head and neck cancer (HNC) remains a significant global health challenge, with nearly 890,000 new cases and approximately 450,000 deaths reported worldwide each year. Radiotherapy is the cornerstone of HNC treatment and it is effective for local disease control;^1^ however, it frequently causes irreversible damage to salivary gland acinar cells,^2^ resulting in salivary hypofunction and a marked reduction in salivary flow rates during the early weeks of treatment.^3^ This reduction in salivary secretion alters both the quantity and composition of saliva^3^ and has been associated with changes in the proteomic profile of the acquired enamel pellicle (AEP).^4^

The AEP is the initial proteinaceous layer that forms on clean enamel surfaces through the selective adsorption of salivary proteins and plays a crucial role in oral homeostasis by providing binding sites for bacterial adhesins, thereby shaping the earliest stages of biofilm formation. Alterations in pellicle composition, such as radiotherapy-induced salivary changes, may influence bacterial adhesion and subsequent biofilm formation.^5,6^

Initial bacterial colonizers, including *Actinomyces*, *Streptococcus*, and *Veillonella* species, selectively adhere to the pellicle and form a substratum that facilitates the introduction and integration of secondary colonizers, shaping the composition and structure of the developing biofilm.^7^ These early colonizers have a profound impact on the microbial ecology of nascent biofilms. For instance, the absence of early colonizers has been shown to alter community structure and quantitative species dynamics in multispecies biofilm models, affecting the abundance and spatial organization of subsequent colonizers.^8^

Disruption of early colonization carries important clinical implications. A stable and balanced early biofilm contributes to oral health by maintaining microbial homeostasis and preventing colonization by opportunistic pathogens. In contrast, disruptions in these early interactions can lead to shifts in microbial succession, predisposing to cariogenic or dysbiotic biofilms and increasing the risk of dental caries and periodontal diseases.^9^ In patients with HNC receiving radiotherapy, radiation-induced salivary changes and concomitant alterations in the pellicle may therefore accelerate dysbiotic shifts in biofilm composition, contributing to the high prevalence of radiation-induced caries reported in this population.^3 4,10,11^

In this context, a better understanding of the earliest bacterial proteins adhering to the AEP is warranted, as these molecules may serve as potential biomarkers for predicting cariogenic shifts or targets for personalized preventive strategies in patients undergoing radiotherapy. Accordingly, the objective of this study was to characterize and compare the bacterial proteomic profiles of early colonizers on the AEP in patients with HNC undergoing radiotherapy, with the goal of elucidating radiation-induced alterations that may affect oral biofilm development and increase the risk of oral health complications. Special emphasis was placed on identifying the pioneer proteins from bacterial species involved in initial adhesion to the AEP.

## Methods

### Ethical aspects and patients

This study was approved by the local Institutional Ethics Committee (process no. 61484116.0.0000.5417). AEPs were collected after institutional ethical approval and signature of an informed consent form by participants. The study was conducted in accordance with the Declaration of Helsinki.

The study population consisted of 18 individuals, including nine patients diagnosed with HNC and nine healthy participants. Sample size was determined based on previous *in vivo* studies involving proteomic analysis of the AEP.^12-17^ Sample size was estimated using MSstats software,^18^ based on data from a previous study conducted by our group.^16^ The significance level was set at α = 0.05, with statistical power (1−β) established at 0.8. An effect size of 1.5 was assumed to account for differences in protein abundance. The calculation indicated that three samples per group were required. Given the limited protein yield typically obtained from *in vivo* AEP samples, nine volunteers were included per group to generate three pooled samples, enabling analyses in biological triplicates.^4^

Patients with HNC were recruited from the Clinical Research Center of the Bauru School of Dentistry. Their ages ranged from 34 to 72 years, and both sexes were represented (seven males and two females). Healthy participants (control group) were selected from the same clinical setting and were matched to patients with HNC according to age (± 5 years) and sex. Sample collection was performed strictly as previously described.^4^

All patients with HNC underwent radiotherapy delivered by a linear accelerator and received irradiation doses of 187 cGy in 33 to 36 treatment sessions.^19^ Tumor sites included oropharyngeal carcinoma and carcinoma of the base of the tongue, occult grade III squamous cell carcinoma with cervical metastasis, squamous cell carcinoma of the tongue, squamous cell carcinoma of the esophagus and palatine tonsil, squamous cell carcinoma of the retromolar trigone (jaw), and hypopharyngeal squamous cell carcinoma. All patients were former smokers and former alcohol users. Any existing oral health issues, such as dental restorations, carious lesions, periodontal disease, or tooth extractions, were addressed prior to radiotherapy.

Inclusion criteria comprised individuals aged over 18 years with a confirmed diagnosis of HNC, patients who required dental treatment prior to AEP collection, patients who had not undergone surgical tumor removal, and those who signed the informed consent form. Exclusion criteria included continued smoking or alcohol use after the diagnosis of HNC. Additionally, during radiotherapy, all patients developed oral mucositis, classified as grade II in four patients, grades II and III in three patients, grade III in one patient, and grades I and II in one patient. Consequently, all participants received laser therapy, as well as analgesic and anti-inflammatory treatment, during radiotherapy.

AEP samples were collected from patients with HNC at three distinct time points: before radiotherapy (BRT), during radiotherapy (DRT; between the second and fifth weeks of treatment), and after radiotherapy (ART; three to four months following completion of therapy). AEP samples for the control group were collected from nine individuals in good systemic and oral health, who were non-smokers, free of dental caries, gingivitis, and periodontitis, or other oral conditions that could influence oral fluid composition, and who were not taking medications or using tobacco products. Participants presenting risk factors for erosive tooth wear, such as high intake of acidic beverages or fruits, frequent swimming, or gastrointestinal conditions including bulimia and gastroesophageal reflux, were excluded from the control group.

### AEP collection

Samples were collected in the morning to minimize possible effects of the circadian rhythm.^20, 21^ Initially, all participants underwent careful dental prophylaxis with prophylactic paste. Thereafter, a 120-minute waiting period was observed to allow for the natural formation of the AEP.

The AEP was collected from all teeth in both dental arches, strictly following the previously described methodology.^17^ Collection was performed after 120 min of pellicle formation, when the pellicle is considered mature and initial bacterial adhesion has already occurred, allowing the evaluation of early biofilm-associated components. Throughout this period, participants were instructed to refrain from eating or drinking. Each quadrant of the dental arches was rinsed with deionized water, air-dried twice, and isolated as much as possible using cotton rolls. The pellicle was obtained using electrode wick filter papers (Bio-Rad, Hercules, CA, USA) measuring 5 × 10 mm, which had been pre-soaked in a 3% citric acid solution. The papers were gently rubbed, without applying pressure, on the coronal two-thirds of the tooth surfaces, avoiding the gingival margin, to prevent contamination. The procedure was performed on the vestibular, lingual, and palatal surfaces.^4^ After collection, the filter papers were placed into 2 mL cryotubes and stored at −80°C until further proteomic analysis.

### Proteomic analysis

Shotgun proteomic analyses were performed exactly as described in previous studies.^4^ For analytical purposes, samples from every three volunteers were combined, resulting in three pooled samples per group and enabling analyses in biological triplicates. Filter papers were cut and placed into individual tubes, one per volunteer. Filter papers were fully immersed in an extraction buffer containing 6 M urea and 2 M thiourea in 50 mM NH_4_HCO_3_ (pH 7.8). The samples were then vortexed for 10 min at 4 °C, sonicated for 5 min, and centrifuged at 20,817 ×g for 10 min at 4C. The supernatants were collected and pooled in groups of three volunteers to obtain biological triplicates. To maximize protein recovery, the extraction procedure was repeated twice. Subsequently, the papers were transferred to filter tubes (Corning® Costar® Spin-X® Plastic Centrifuge Tube Filters) and centrifuged again at 20,817 × g for 10 min at 4 °C. The filtrate was recovered and added to the previously collected supernatants. The samples were then centrifuged once more under the same conditions, and the resulting supernatant was collected. Subsequently, 1.5 volumes of 50 mM NH_4_HCO_3_ were added, based on the total sample volume, to reduce the concentrations of urea and thiourea, which could interfere with trypsin activity. The samples were transferred to Amicon Ultra-15 centrifugal filter units (Merck Millipore®, Tullagreen, County Cork, Ireland) and centrifuged at 4,500 ×g at 4°C until a final volume of approximately 100 µL was obtained. Total protein concentration was determined using the Bradford assay (Bio-Rad Bradford Assays, Hercules, USA). Protein reduction was achieved by adding 5 mM dithiothreitol (DTT) followed by incubation at 37°C for 40 min. Alkylation was then carried out with 10 mM iodoacetamide (GE Healthcare, Little Chalfont, Buckinghamshire, UK) for 30 min in the absence of light. After protein reduction and alkylation, 100 µL of 50 mM NH_4_HCO_3_ (pH 7.8) was added, and tryptic digestion was conducted for 14 h at 37°C using trypsin (Thermo Scientific Pierce Trypsin Protease, Rockford, USA). Enzymatic digestion was terminated by the addition of 5% formic acid, followed by desalting and purification using C18 spin columns (Thermo Scientific, Rockford, USA). A 1 µL aliquot from each sample was collected for post-digestion protein quantification using the Bradford method. After quantification, samples were resuspended in 3% acetonitrile containing 0.1% formic acid and subsequently prepared for nanoLC-ESI-MS/MS analysis.

### NanoLC-ESI-MS/MS acquisition

Peptides were analyzed on a Xevo G2 mass spectrometer (Waters) coupled to a nanoACQUITY system (Waters). All samples were analyzed in technical triplicates. Mass spectrometer operational parameters were detailed in a previous study.^17^ Data processing and protein identification from continuous LC-MSE runs were conducted using ProteinLynx Global Server (PLGS) software, version 3.0. Proteins were identified using the software’s ion-counting algorithm, and a search was performed against the oral bacterial database (UniProtKB/Swiss-Prot; http://www.uniprot.org).

### Shotgun label-free quantitative proteomic analysis

Label-free quantitative proteomic analysis was conducted using PLGS software (version 3.0, Waters, Manchester, UK). Three raw MS files from each experimental group were included. Only proteins identified with a confidence interval greater than 95% were considered for quantitative analysis. Identical peptides detected across technical triplicates were grouped based on mass accuracy (< 10 ppm) and retention time tolerance (<0.25 min) using the clustering tool within PLGS. The difference in protein expression between the groups was analyzed by the *t* test (p < 0.05). Proteins were considered downregulated when p<0.05 and upregulated when 1−p > 0.95. The following comparisons were performed: BRT versus Control; DRT versus BRT; BRT versus ART. Protein identification and data organization for bioinformatics analysis were performed using the UNIPROT database (http://www.uniprot.org), including both reviewed and unreviewed entries. Reverse sequences, duplicate proteins, and repeated fragments were excluded from the analysis.

## Results

Table 1 presents the relative quantification of bacterial proteins identified in the AEP for the comparison between BRT and control. Six bacterial proteins were significantly more abundant BRT than in the control group, with increases greater than twofold. These proteins were predominantly derived from *Actinomyces* species and were mainly associated with glucose metabolism and DNA structural organization. *Glyceraldehyde-3-phosphate dehydrogenase* showed the highest increase in abundance at BRT, yielding a 3.56-fold increase; p < 0.01), followed by several histone-like DNA-binding proteins (HB1 and HU family proteins), all of which exhibited threefold increases. These proteins are involved in DNA binding, chromatin structuring, and chromosomal condensation. In contrast, multiple proteins were exclusively detected in the control group, including enzymes involved in methyltransferase activity, DNA replication initiation, tRNA modification, and several uncharacterized proteins, mainly from *Capnocytophaga*, *Prevotella*, and *Olsenella* species. *Cysteine synthase* from *Actinomyces* sp. was uniquely identified BRT.

**Table 1:** Relative quantification of bacterial proteins identified on acquired enamel pellicle from healthy patients (control) and patients with head and neck cancer before radiotherapy (BRT).

Access number	Protein name*	PLGS score	Ratio	p-values	Protein function**
			BRT:Control		
L1PFP6	Glyceraldehyde-3-phosphate dehydrogenase OS=Actinomyces sp. oral taxon 181 str. F0379	455	3.56	< 0.01	Glucose metabolic process;
					glyceraldehyde-3-phosphate dehydrogenase (NAD+) activity (phosphorylation);
					NAD binding; NADP binding
E7NDS1	DNA-binding protein HB1 OS=Actinomyces sp. oral taxon 171 str. F0337	696	3.13	< 0.01	DNA binding;
					structural component of chromatin
F3PBV0	DNA-binding protein HU-alpha OS=Actinomyces sp. oral taxon 170 str. F0386	696	3.13	0.02	DNA binding; structural component of chromatin
G9PMW1	DNA-binding protein HB1 OS=Actinomyces sp. oral taxon 849 str. F0330	696	3.06	< 0.01	DNA binding, structural component of chromatin
F9EHV8	DNA-binding protein HU OS=Actinomyces sp. oral taxon 448 str. F0400	696	3.00	< 0.01	DNA binding; structural component of chromatin
A0A2S0LKX0	Integration host factor OS=Actinomyces sp. oral taxon 897	696	2.94	< 0.01	DNA binding; structural component of chromatin; chromosomal condensation
F9PMQ7	Cysteine synthase OS=Actinomyces sp. oral taxon 175 str. F0384	BRT*	-	-	Cysteine synthase activity;
					cysteine biosynthesis from serine
D3IAR7	dTDP-4-dehydrorhamnose 3_5-epimerase OS=Prevotella sp. oral taxon 299 str. F0039	Control*	-	-	Dtdp-4-dehydroramnose 3,5-epimerase; dTDP-rhamnose biosynthetic process
I8UL97	Methyltransferase domain protein OS=Capnocytophaga sp. oral taxon 412 str. F0487	Control*	-	-	Methyltransferase activity dependent on S-adenosylmethionine; methylation
A0A2S0L8X1	Methyltransferase OS=Capnocytophaga sp. oral taxon 878	Control*	-	-	Methyltransferase activity dependent on S-adenosylmethionine; methylation
D3IGK9	Replication initiation protein OS=Prevotella sp. oral taxon 317 str. F0108	Control*	-	-	Replication initiation proteins are necessary to open the double DNA helix and initiate DNA replication in bacteria
A0A2S0L935	tRNA-specific 2-thiouridylase MnmA OS=Capnocytophaga sp. oral taxon 878	Control*	-	-	Catalytic activity mediating the unidirectional 2-thiolation of the positive swing (U34) of tRNA, leading to the formation of s2U34
L1PPF2	Uncharacterized protein OS=Capnocytophaga sp. oral taxon 324 str. F0483	Control*	-	-	Uncharacterized protein
L1PDJ0	Uncharacterized protein OS=Capnocytophaga sp. oral taxon 324 str. F0483	Control*	-	-	Activity of methyltransferase dependent on S-adenosylmethionine
L1Q0W6	Uncharacterized protein OS=Capnocytophaga sp. oral taxon 326 str. F0382	Control*	-	-	Activity of methyltransferase dependent on S-adenosylmethionine
S3BMC1	Uncharacterized protein OS=Capnocytophaga sp. oral taxon 336 str. F0502	Control*	-	-	Uncharacterized protein
S3BHQ6	Uncharacterized protein OS=Capnocytophaga sp. oral taxon 336 str. F0502	Control*	-	-	Activity of methyltransferase dependent on S-adenosylmethionine
A0A0K1F0W8	Uncharacterized protein OS=Olsenella sp. oral taxon 807	Control*	-	-	Uncharacterized protein
A0A2T4TJP5	Uncharacterized protein OS=Prevotella sp. oral taxon 313	Control*	-	-	Uncharacterized protein

*Protein function are based on protein ID from UniProt protein database (http://wwwwww.uniprot.org/); ^**^Proteins with significantly altered expression are organized according to the ratio. ^*^Indicates unique proteins in alphabetical order. Proteins in bold are increased more than twofold.

In the comparison between BRT and DRT, proteins uniquely detected in the BRT period were mainly associated with DNA binding and chromatin structure, including histone-like proteins (HB1 and HU family) and *Cysteine synthase*. These findings suggest a predominance of proteins involved in genomic organization and basic cellular maintenance prior to radiotherapy. A marked shift in the diversity of bacterial proteins was observed DRT. Proteins uniquely identified DRT were predominantly involved in carbohydrate transport and metabolism, stress response, redox balance, and protein folding. Many ATP-binding cassette (ABC) transporter proteins, originating from diverse bacterial genera, were detected, indicating enhanced transport activity DRT. In addition, glycolytic enzymes, such as *Fructose-bisphosphate aldolase* and *Lactate dehydrogenase*, were exclusively found DRT. Proteins associated with oxidative stress control, including *Thioredoxin reductase*, and molecular chaperones (e.g., 60 kDa chaperonin), were also identified only DRT. Several uncharacterized proteins from *Tannerella*, *Prevotella*, and *Selenomonas* species were also detected exclusively DRT. When BRT and DRT were compared, a subset of bacterial proteins showed no significant changes in expression throughout the radiotherapy period. Their abundance remained stable during treatment, with no evidence of statistically significant upregulation or downregulation (p > 0.05) (Table 2).

**Table 2:** Bacterial proteins identified on acquired enamel pellicle from patients with head and neck cancer before (BRT) and during (DRT) radiotherapy.

Access number	Protein name	PLGS score	Unique*	p-value	Function
F9PMQ7	Cysteine synthase OS=Actinomyces sp. oral taxon 175 str. F0384	91	BRT	NC	Cysteine synthase activity, cysteine biosynthesis from serine
D0WNJ5	DNA-binding protein HB1 OS=Actinomyces sp. oral taxon 848 str. F0332	987	BRT	NC	DNA binding, structural constituent of chromatin
G9PMW1	DNA-binding protein HB1 OS=Actinomyces sp. oral taxon 849 str. F0330	987	BRT	NC	DNA binding, structural constituent of chromatin
F9EHV8	DNA-binding protein HU OS=Actinomyces sp. oral taxon 448 str. F0400	987	BRT	NC	DNA binding, structural constituent of chromatin
F3PBV0	DNA-binding protein HU-alpha OS=Actinomyces sp. oral taxon 170 str. F0386	987	BRT	NC	DNA binding, structural constituent of chromatin
A0A0M4GFM1	60 kDa chaperonin OS=Actinomyces sp. oral taxon 414	127	DRT	NC	Capsule, cell surface, cytoplasm, ATP binding, ATP-dependent protein folding companion, isomerase activity, unfolded protein binding, protein doubling
A0A2T4T1K6	3-deoxy-D-manno-octulosonate 8-phosphate phosphatase OS=Prevotella sp. oral taxon 820	137	DRT	NC	Hydrolase activity, acting on ester bonds, metal ion binding
G9PJA1		80	DRT	NC	ATP binding carrier complex (ABC), ABC-type transporter activity, ATP binding, ATP hydrolysis activity and carbohydrate transport
	ABC transporter domain-containing protein OS=Actinomyces sp. oral taxon 849 str. F0330				
G5F356	ABC transporter domain-containing protein OS=Olsenella sp. oral taxon 809 str. F0356	80	DRT	NC	ATP binding carrier complex (ABC), ABC-type transporter activity, ATP binding, ATP hydrolysis activity, and carbohydrate transport
U2EIT0	ABC transporter_ ATP-binding protein OS=Clostridiales bacterium oral taxon 876 str. F0540	80	DRT	NC	ATP binding carrier complex (ABC), ABC-type transporter activity, ATP binding, ATP hydrolysis activity, and carbohydrate transport
H1LYP5	ABC transporter_ ATP-binding protein OS=Lachnospiraceae bacterium oral taxon 082 str. F0431	80	DRT	NC	ATP binding carrier complex (ABC), ABC-type transporter activity, ATP binding, ATP hydrolysis activity, and carbohydrate transport
F5T447	ABC transporter_ ATP-binding protein OS=Oribacterium sp. oral taxon 108 str. F0425	80	DRT	NC	ATP binding carrier complex (ABC), ABC-type transporter activity, ATP binding, ATP hydrolysis activity, and carbohydrate transport
E0Q2F5	ABC transporter_ ATP-binding protein OS=Streptococcus sp. oral taxon 071 str. 73H25AP	80	DRT	NC	ATP binding cassette carrier complex (ABC), ABC type transporter activity, ATP binding, ATP hydrolysis activity, and carbohydrate transport
L1PB56	Conjugative transposon protein TraI OS=Capnocytophaga sp. oral taxon 324 str. F0483	69	DRT	NC	Conjugative Transposon Protein TraI
A0A0M4H7M4	Fructose-1_6-bisphosphate aldolase OS=Actinomyces sp. oral taxon 414	199	DRT	NC	Fructose-biphosphate aldolase activity, glycolytic process
F9EDG0	Fructose-bisphosphate aldolase OS=Actinomyces sp. oral taxon 448 str. F0400	200	DRT	NC	Fructose-biphosphate aldolase activity, glycolytic process
A0A0M4GT11	L-lactate dehydrogenase OS=Actinomyces sp. oral taxon 414	179	DRT	NC	Cytoplasm, L-lactate dehydrogenase activity, glycolytic process
A0A2S0LU24	Lipocalin OS=Dietzia sp. oral taxon 368	121	DRT	NC	Proteins that bind to small hydrophobic molecules, such as lipids and retinoids. They transport lipids, vitamins, hormones, and metabolites. Involved in immune responses, cellular migration and differentiation. Associated with the interactions of cancer cells and biological synthesis of prostaglandins.
F3B1Q7	Maltodextrin import ATP-binding protein msmX OS=Lachnospiraceae oral taxon 107 str. F0167	80	DRT	NC	ATP binding cassette carrier complex (ABC), ABC type transporter activity, ATP binding, ATP hydrolysis activity, and carbohydrate transport
S2ZT92	Maltose/maltodextrin transport system ATP-binding protein OS=Atopobium sp. oral taxon 199 str. F0494	80	DRT	NC	ATP binding cassette carrier complex (ABC), ABC type transporter activity, ATP binding, ATP hydrolysis activity, and carbohydrate transport
A0A1B3X4Z9	Mannitol-1-phosphate 5-dehydrogenase OS=Selenomonas sp. oral taxon 920	112	DRT	NC	Mannitol-1-phosphate 5-dehydrogenase activity, mannitol metabolic process
L1PRA4	Nitroreductase family protein OS=Capnocytophaga sp. oral taxon 324 str. F0483	80	DRT	NC	Oxidoreductase activity
A0A2R3N0E1	Sugar ABC transporter ATP-binding protein OS=Lachnospiraceae bacterium oral taxon 500	80	DRT	NC	ATP binding carrier complex (ABC), ABC-type transporter activity, ATP binding, ATP hydrolysis activity, and carbohydrate transport
A0A0K1F3A3	Sugar ABC transporter ATP-binding protein OS=Olsenella sp. oral taxon 807	80	DRT	NC	ATP binding carrier complex (ABC), ABC-type transporter activity, ATP binding, ATP hydrolysis activity, and carbohydrate transport
A0A0X8JXI3	Sugar ABC transporter ATP-binding protein OS=Streptococcus sp. oral taxon 431	80	DRT	NC	ATP binding carrier complex (ABC), ABC-type transporter activity, ATP binding, ATP hydrolysis activity, and carbohydrate transport
F9EDZ0	Sugar ABC superfamily ATP binding cassette transporter_ ABC protein OS=Actinomyces sp. oral taxon 448 str. F0400	111	DRT	NC	ATP binding carrier complex (ABC), ABC-type transporter activity, ATP binding, ATP hydrolysis activity, and carbohydrate transport
A0A0M4GDJ0	Sugar ABC transporter ATP-binding protein OS=Actinomyces sp. oral taxon 414	111	DRT	NC	ATP binding carrier complex (ABC), ABC-type transporter activity, ATP binding, ATP hydrolysis activity, and carbohydrate transport
F9ED26	Thioredoxin reductase OS=Actinomyces sp. oral taxon 448 str. F0400	163	DRT	NC	Cytoplasm, thioredoxine-disulfide reductase (NADP) activity, removal of superoxide radicals
F9HGK8	TOBE domain protein OS=Streptococcus sp. oral taxon 056 str. F0418	80	DRT	NC	ATP binding carrier complex (ABC), ABC-type transporter activity, ATP binding, ATP hydrolysis activity, and carbohydrate transport
A0A2N8NJX9	Uncharacterized protein OS=Tannerella sp. oral taxon 808	40	DRT	NC	Uncharacterized protein
W2CLQ4	Uncharacterized protein OS=Tannerella sp. oral taxon BU063 isolate Cell 6/7/9	56	DRT	NC	Uncharacterized protein
D3IJU8	Uncharacterized protein OS=Prevotella sp. oral taxon 317 str. F0108	80	DRT	NC	Uncharacterized protein
U2M781	Uncharacterized protein OS=Selenomonas sp. oral taxon 892 str. F0426	139	DRT	NC	Uncharacterized protein

Identification and protein function are based on protein ID from UniProt protein database (http://wwwwww.uniprot.org/). ^*^Indicates unique proteins in alphabetical order. NC (not computed) indicates no differences in expression.

Similarly, the comparison between BRT and ART revealed no significant differences in the expression of several bacterial proteins. These proteins exhibited comparable abundance levels before and after radiotherapy, suggesting maintenance of their expression profiles over time (p > 0.05). Proteins uniquely identified BRT were again predominantly related to DNA binding and chromatin organization, including multiple histone-like proteins and integration host factors from *Actinomyces* species, as well as *Cysteine synthase*. In contrast, ART was characterized by the exclusive presence of proteins associated with stress tolerance, transcriptional regulation, and secretion systems. Notably, several C-terminal sorting domain-containing proteins belonging to secretion systems of *Tannerella* species were detected only ART. Additionally, proteins involved in cellular protection against environmental stress, such as *Late embryogenesis abundant*, and *Transcriptional regulator*_XRE family were identified exclusively ART. These findings indicate a shift in the bacterial proteomic profile on the AEP after radiotherapy, characterized by the enrichment of proteins potentially involved in adaptation to stress and changes in the oral environment (Table 3).

**Table 3:** Bacterial proteins identified on acquired enamel pellicle from patients with head and neck cancer before (BRT) and after (ART) radiotherapy.

Access number	Protein name	PLGS score	*Unique	p-value	^b^Function
F9PMQ7	Cysteine synthase OS=Actinomyces sp. oral taxon 175 str. F0384	91	BRT	NC	Cysteine synthase activity, cysteine biosynthesis from serine
F9PJ25	DNA-binding protein HB1 OS=Actinomyces sp. oral taxon 175 str. F0384	987	BRT	NC	Histone-like DNA-binding protein capable of wrapping around DNA to stabilize it and thus prevent its denaturation under extreme environmental conditions
F9EHV8	DNA-binding protein HU OS=Actinomyces sp. oral taxon 448 str. F0400	987	BRT	NC	DNA binding, structural constituent of chromatin
F3PBV0	DNA-binding protein HU-alpha OS=Actinomyces sp. oral taxon 170 str. F0386	987	BRT	NC	DNA binding, structural constituent of chromatin
G9PMW1	DNA-binding protein HB1 OS=Actinomyces sp. oral taxon 849 str. F0330	987	BRT	NC	DNA binding, structural constituent of chromatin
A0A0M4H4B4	Integration host factor OS=Actinomyces sp. oral taxon 414	987	BRT	NC	DNA binding, structural constituent of chromatin
U2AR85	DUF1963 domain-containing protein OS=Capnocytophaga sp. oral taxon 863 str. F0517	116	ART	NC	DUF1963 domain-containing proteins that are basic units that can fold, function, and evolve independently
U2W4E1	Late embryogeneis abundant protein OS=Oribacterium sp. oral taxon 078 str. F0263	111	ART	NC	They protect cells from damage caused by environmental stresses, such as changes in temperature, salinity, and dryness, by providing buffer hydration.
W2CIT0	Secretion system C-terminal sorting domain-containing protein OS=Tannerella sp. oral taxon BU063 isolate Cell 1/3	114	ART	NC	C-terminal sorting domain-containing protein of the secretion system
W2C4L3	Secretion system C-terminal sorting domain-containing protein OS=Tannerella sp. oral taxon BU063 isolate Cell 2	114	ART	NC	C-terminal sorting domain-containing protein of the secretion system
W2CA78	Secretion system C-terminal sorting domain-containing protein OS=Tannerella sp. oral taxon BU063 isolate Cell 5	114	ART	NC	C-terminal sorting domain-containing protein of the secretion system
W2CMU8	Secretion system C-terminal sorting domain-containing protein OS=Tannerella sp. oral taxon BU063 isolate Cell 6/7/9	116	ART	NC	C-terminal sorting domain-containing protein of the secretion system
W2CY98	Secretion system C-terminal sorting domain-containing protein OS=Tannerella sp. oral taxon BU063 isolate Cell 8/11	114	ART	NC	C-terminal sorting domain-containing protein of the secretion system
D7JDQ2	Transcriptional regulator_ XRE family OS=Bacteroidetes oral taxon 274 str. F0058	605	ART	NC	DNA binding

Identification and protein function are based on protein ID from UniProt protein database (http://wwwwww.uniprot.org/). ^*^Indicates unique proteins in alphabetical order. NC (not computed) indicates no differences in expression.

## Discussion

Significant alterations were observed in the AEP bacterial proteomic profile in patients with HNC BRT, DRT, and ART, as well as in the comparison with the control. To the best of our knowledge, this is the first study to evaluate bacterial proteomic composition at an early stage after AEP formation using molecular tools such as proteomic analysis.

In microbial biofilms, such as dental plaque, the extracellular matrix is a critical element, as it maintains the cellular spatial organization and coordinates cellular functions within the biofilm structure. The extracellular polymeric matrix is composed of carbohydrates, nucleic acids, proteins, and lipids, which are often organized into macromolecular complexes and/or associated with microbial cell surfaces within the biofilm.^22^ Therefore, strategies for understanding the proteomic profile and matrix control are essential for maintaining oral health, particularly in patients with HNC undergoing radiotherapy, with direct implications for their quality of life.

In this context, comparison of bacterial proteins between patients with HNC BRT and control individuals revealed a pronounced increase (more than twofold) in key proteins involved in oral health homeostasis. Multiple isoforms of the *D*NA-binding protein and Integration host factor, both from *Actinomyces sp. oral taxon,* were among the most prominent. These proteins are associated with DNA binding and chromatin structure (UNIPROT). Additionally, the levels of proteins involved in glucose metabolism and phosphorylation, such as *Glyceraldehyde-3-phosphate dehydrogenas*e from *Actinomyces sp. Oral taxon*, were more elevated in patients with cancer than in control individuals (Table 1).

Proteins involved in glucose metabolism were also found exclusively DRT when comparing bacterial proteins from patients with HNC before and during radiotherapy. Notably, such proteins included *Fructose-1,6-bisphosphate aldolase, Lipocalin, Nitroreductase family protein, Maltodextrin import ATP-binding protein, Maltose/maltodextrin transport system ATP-binding protein, Mannitol-1-phosphate 5-dehydrogenase, L-lactate dehydrogenase*, and *Sugar ABC transporter ATP-binding protein* from different bacterial species (Table 2). This finding is particularly noteworthy given that cariogenic dental plaque is rich in glucans and fructans derived from microbial extracellular metabolism of dietary sucrose.^22^ In this regard, the proteomic profile at the early stages of dental plaque formation in patients with cancer differs from that of healthy individuals and undergoes some changes during radiotherapy, demonstrating an increase in proteins potentially involved in glucose metabolism.

Furthermore, different proteins were identified in several bacterial species primarily associated with periodontitis and gingivitis ART when compared with BRT. These proteins include *Oribacterium sp.*, *oral taxon* ^23^ *Tannerella sp.*, *oral taxon* ^24^ and *Bacteroidetes, oral taxon*,^25^ which are associated with *Late embryogenesis abundant protein, Secretion system C-terminal sorting domain-containing protein*, and *Transcriptional regulator (XRE family)*, respectively (Table 3). Studies have demonstrated that salivary flow is markedly reduced after radiotherapy,^3^ resulting in decreased levels of essential proteins for the protection of dental enamel^4^ and the oral cavity.^3^ This reduction in immune defense may facilitate the initial colonization of the dental biofilm by these pathogenic bacteria.^26^ The identification of these bacterial proteins may also be correlated with the smoking history of the analyzed individuals, as smoking has been shown to be a key factor in modifying the oral microbiome.^23^

Finally, *Cysteine synthase* was identified exclusively in patients with cancer BRT in all comparisons. This protein is involved in cysteine biosynthesis from serine (UNIPROT). Nevertheless, recent studies have shown that *Cysteine synthase* is considered a “*moonlighting*” protein, performing functions that extend beyond those previously ascribed to it. In addition to catalyzing cysteine synthesis, *Cysteine synthase* plays non-canonical regulatory functions by binding to and modulating the activity of various proteins. Apart from its catalytic and regulatory role within the cysteine biosynthesis pathway, *Cysteine synthase* exerts a “hidden” effect by binding to other proteins containing a C-terminal “CS-binding motif” ending in a terminal isoleucine.^27^ In this context, this protein could be a strong candidate as a biomarker for HNC, as it was identified exclusively in this group of patients; however, definitive conclusions cannot be drawn due to the small sample size and the need for further confirmatory tests. Thus, our findings may provide a foundation for future studies involving this important approach.

A limitation of the present study is the necessity for supportive clinical care during radiotherapy in patients with HNC. Given the high incidence of moderate-to-severe oral mucositis, all patients received photobiomodulation (laser therapy) and pharmacological management with analgesic and anti-inflammatory drugs as part of routine and ethical clinical care. Therefore, the effects of these interventions on the proteomic profile cannot be completely ruled out. Consequently, the observed proteomic alterations should be interpreted in light of this inherent limitation, which reflects real-world clinical care and underscores the translational relevance of the findings.

## Conclusion

In conclusion, this study provides novel insights into the dynamic alterations of the bacterial proteomic profile of the AEP in patients with HNC undergoing radiotherapy. The identification of distinct protein signatures associated with early bacterial colonizers across different stages of radiotherapy highlights molecular pathways potentially involved in biofilm dysbiosis and increased susceptibility to oral diseases. Importantly, the detection of specific bacterial proteins exhibiting exclusive or differential expression patterns suggests their potential as early biomarkers for radiation-induced oral complications. These findings help elucidate host-microbial interactions under radiotherapy-induced stress and may support the development of targeted preventive and personalized oral care strategies aimed at preserving oral health and improving quality of life in patients with HNC.

## Data Availability

The authors declare that all data generated or analyzed during this study are included in this published article.
